# HOW REAL IS THIS DEAL? EVALUATING AI ACCURACY IN PROVIDING SUPPORT TO DEMENTIA CAREGIVERS

**DOI:** 10.1093/geroni/igad104.3399

**Published:** 2023-12-21

**Authors:** Jeanette Ross, Elizabeth Reilly, Sandra Sanchez-Reilly, Lyda Arevalo-Flechas, Alexander Dentchev

**Affiliations:** South Texas Veterans Health Care System, San Antonio, Texas, United States; Health Careers High School, San Antonio, Texas, United States; South Texas Veterans Health Care System, San Antonio, Texas, United States; South Texas Veterans Health Care System, San Antonio, Texas, United States; Saint Mary’s Hall, San Antonio, Texas, United States

## Abstract

**Background:**

Dementia, a devastating and incurable disease, affects millions of older Americans, placing astronomical levels of burden on caregivers, who usually lack resources to learn to provide their loved ones with high-quality care. Artificial Intelligence (AI), a programmed pioneering resource to generate immediate automated responses, is universally available when one looks for potentially accurate answers. Objective: To evaluate accuracy of AI sites pertaining to questions related to dementia caregiving

**Methods:**

AI sites (ChatGPT and Textero.ai) were asked questions about dementia caregiving aspects such as self-care, patient symptoms/complications, stress control and managing expectations. Dementia caregiving experts reviewed and classified responses using the validated Zarit Caregiver Burden Scale (ZCBS) as framework. Inter-reliability scores were calculated among reviewers’ responses.

**Results:**

AI sites responded in paragraph and list formats. Common themes identified by reviewers from both AI sites included self-care suggestions and seeking/accepting help. Several ZCBS aspects were not addressed by both sites such as guilt, exhaustion, negative emotions, and patient’s dependence. Textero.ai obtained lower scores addressing ZCBS, specifically not addressing potential caregiver’s unreasonable expectations. Inter-reliability score (reviewers) was 0.91 (highly significant).

**Conclusions:**

In evaluating varied aspects of caregiving, results showed that AI sites, as it pertains to dementia caregiving are promising resources for accurately emphasizing caregiver’s self-help encouraging positive thoughts. However, validation and exploration of negative feelings and emotions within AI sites might not be properly addressed for dementia caregivers. Further studies need to further explore the use of AI in combination with hands-on dementia interventions to validate all aspects of caregiving.

